# Age-Metabolic Profile of Lower Serum Estradiol Among Women in Kazakhstan: A Large National Survey-Based Analytical Study

**DOI:** 10.3390/ijerph23070934

**Published:** 2026-07-21

**Authors:** Bolat Sadykov, Anel Ibrayeva, Yerlan Ismoldayev, Marat Shoranov, Timur Saliev, Altynay Sadykova, Shynar Tanabayeva, Ildar Fakhradiyev

**Affiliations:** 1 Department of Medicine, S.D. Asfendiyarov Kazakh National Medical University, Almaty 050000, Kazakhstan; 2 College of Medicine, Korea University, Seoul 02841, Republic of Korea

**Keywords:** estradiol, women, Kazakhstan, population-based study, hormones, cross-sectional survey, adiposity, HDL cholesterol, agemetabolic profile

## Abstract

**Highlights:**

**Public health relevance—How does this work relate to a public health issue?**

- Lower serum estradiol values in women may reflect not only reproductive aging but also broader age–metabolic health patterns relevant to noncommunicable disease prevention.
- This national survey-based analytical sample provides rare survey-based evidence on serum estradiol distribution among adult women in Kazakhstan.

**Public health significance—Why is this work of significance to public health?**

- In this analytical sample, the lower-tail estradiol category was mainly associated with age, overweight/obesity, and low HDL cholesterol rather than stable regional clustering.
- These findings help shift interpretation from geographic differences toward individual cardiometabolic and reproductive-aging profiles.

**Public health implications—What are the key implications or messages for practitioners, policy makers and/or researchers in public health?**

- These findings may inform future epidemiological research on reproductive-aging and metabolic profiles, but do not support general-population estradiol screening for metabolic risk stratification.
- Future public health research should include menstrual-cycle timing, menopausal status, hormonal medication use, pregnancy and lactation status, and repeated hormone measurements to improve clinical interpretation.

**Abstract:**

Serum estradiol is a central biomarker of female reproductive biology and is also linked to bone, metabolic, and cardiovascular health. Population-level data on estradiol distribution among women in Kazakhstan remain limited, and it is unclear whether lower estradiol categories in national survey data reflect geographic clustering or individual age-metabolic composition. We performed a secondary analysis of a large national survey-based analytical sample from Kazakhstan using the WHO STEPS framework. The analytical sample included 3498 women aged 18–69 years with available serum estradiol measurements and core covariate data. The primary binary outcome was a lower-tail estradiol category defined as E2 < 200 pmol/L, corresponding approximately to the empirical lower quartile of the analytical female subsample. Descriptive statistics, age-specific quartile summaries, multivariable logistic regression, continuous-outcome regression for ln(E2), nonlinearity diagnostics, and regional heterogeneity analyses were used. Median serum estradiol was 313.00 pmol/L (interquartile range 207.25–457.00). In unweighted and unadjusted analytical-sample comparisons, there was little evidence of regional heterogeneity in estradiol distribution (ANOVA *p* = 0.583; Kruskal–Wallis *p* = 0.287; eta-squared = 0.0049; ICC for ln[E2] = 0.000861). In the multivariable model, compared with women aged 18–29 years, the odds of belonging to the lower-tail estradiol category increased from the 40–49-year group (aOR 1.82, 95% CI 1.31–2.53) to the 50–59-year group (aOR 3.72, 95% CI 2.60–5.33) and were highest in the 60–69-year group (aOR 7.48, 95% CI 5.23–10.70). Overweight and obesity were associated with higher odds of lower-tail estradiol, and low HDL cholesterol showed a modest independent association (aOR 1.26, 95% CI 1.04–1.53). In this large national survey-based analytical sample of women, lower-tail estradiol was more closely characterized by age, adiposity, and HDL cholesterol than by regional clustering. The lower estradiol category used in this study should not be interpreted as a clinical diagnosis of hypoestrogenism, particularly because reproductive-stage information was unavailable.

## 1. Introduction

Serum estradiol (E2) is the main and most biologically active estrogen in women and plays a central role in folliculogenesis, menstrual cyclicity, endometrial function, and reproductive aging [1]. Beyond the reproductive axis, estradiol is involved in skeletal homeostasis, vascular biology, glucose regulation, and lipid metabolism [2,3,4]. Serum concentrations vary substantially across the life course and are shaped by ovarian reserve, menopausal transition, adiposity, and broader metabolic context [5,6].

From a clinical and epidemiological perspective, the lower tail of the estradiol distribution is of particular interest because it may reflect reproductive aging as well as broader physiological states linked to bone [7], metabolic [8] and cardiovascular health [9]. At the same time, interpretation of estradiol values in population studies is challenging because hormone levels are strongly influenced by age, phase of the menstrual cycle, menopausal status, pregnancy and lactation, hormonal medication use, and assay characteristics [10].

The STRAW + 10 framework conceptualizes reproductive aging as a staged process based on menstrual-cycle characteristics and endocrine markers, including FSH, AMH, inhibin B, and antral follicle count, rather than chronological age alone [11]. Because the present survey did not include menstrual bleeding patterns, FSH, AMH, inhibin B, or antral follicle count, age was used only as an epidemiological descriptor and an imperfect proxy for reproductive stage, not as a direct clinical measure of reproductive aging.

Although international evidence on steroid hormone reference distributions has expanded in recent years, most published data come from specific populations and cannot be transferred directly across settings with different age structures, ethnic composition, lifestyles, and burdens of cardiometabolic risk, which may influence estradiol distribution through metabolic pathways [12]. For Kazakhstan, published studies relevant to sex hormones and metabolic risk have largely focused on narrower populations, including women with polycystic ovary syndrome and reproductive-age women with metabolic syndrome [13,14]; population-level evidence on estradiol distribution among adult women in Kazakhstan remains limited.

The geographic dimension is also analytically important. Kazakhstan covers a large territory with marked regional differences in urbanization, lifestyle, socioeconomic context, and population structure. However, apparent regional differences in a hormone measured once in a cross-sectional survey may reflect the age and metabolic composition of regional subsamples rather than stable geographic clustering. This distinction matters because an overemphasis on geography could obscure the more clinically and epidemiologically relevant individual-level profile.

Therefore, the present study aimed to describe serum estradiol distribution in a large national survey-based analytical sample of adult women in Kazakhstan and to examine demographic, anthropometric, behavioral, and metabolic factors associated with the lower tail of the estradiol distribution. Because a single universal clinical threshold is not appropriate for women aged 18–69 years, the binary outcome was defined pragmatically as E2 < 200 pmol/L, a rounded and interpretable approximation of the empirical lower quartile of the analytical female subsample. This threshold was used as a statistical categorization of the lower part of the observed distribution rather than as a stand-alone clinical diagnosis.

## 2. Materials and Methods

### 2.1. Study Design and Data Source

This study is a secondary analysis of data from a nationwide cross-sectional survey of adults in Kazakhstan conducted using the WHO STEPwise approach to noncommunicable disease risk factor surveillance (WHO STEPS) [15]. The survey included questionnaire-based interviews, physical examinations, and biochemical measurements. For the present analysis, women with available serum estradiol measurements and relevant covariates were selected. The survey was carried out between May and October 2025 across all administrative-territorial units of Kazakhstan, including 17 regions and 3 cities of republican significance.

### 2.2. Sampling and Study Population

The parent survey provided the source population for a large national survey-based analytical sample of adults aged 18–69 years and included 6720 participants before selection of the female estradiol subsample. Sampling followed a multistage stratified cluster design. At the first stage, the sample was stratified by sex and age group. Subsequent stages included selection of districts and cities, then primary healthcare organizations and households. One respondent per household was selected using the Kish method.

**Figure 1:**
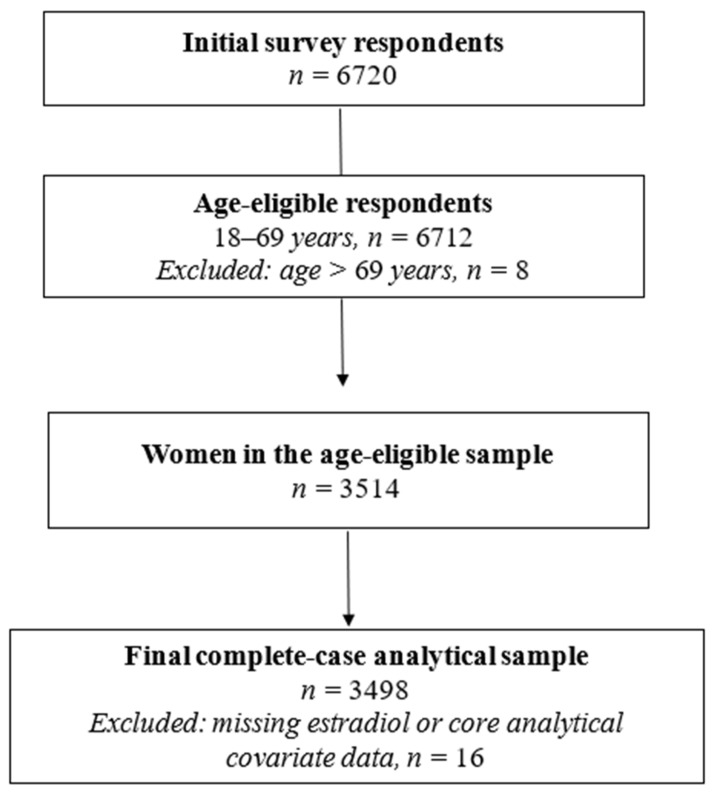
Flow diagram of analytical sample selection.

The derivation of the final analytical sample is presented in Figure 1. The initial survey included 6720 respondents, of whom 6712 were within the eligible age range of 18–69 years. Among age-eligible respondents, 3514 were women. After exclusion of 16 women with missing serum estradiol measurements or incomplete core analytical covariate data, 3498 women were included in the final complete-case analytical sample. The age range of the analytical sample reflected the design of the parent survey. In the absence of reproductive-stage data, age groups were interpreted as epidemiological strata rather than clinical categories of reproductive or menopausal status.

### 2.3. Data Collection and Laboratory Measurements

Data were collected by trained field teams according to standardized STEPS procedures. During a single survey contact, participants underwent an interview, anthropometric assessment, blood pressure measurement, and venous blood sampling for biochemical analyses.

Anthropometric assessment included measurement of height and body weight followed by calculation of body mass index (BMI, kg/m^2^) [16]. Questionnaire variables included place of residence, education, ethnicity, marital status, smoking status, and alcohol consumption during the previous 12 months. The biochemical panel included glycated hemoglobin (HbA1c) and lipid parameters.

Serum estradiol was measured by INVITRO Kazakhstan using the Estradiol assay, article/test code 62. According to the laboratory report template, the assay was performed on the Abbott Alinity i analytical platform using the Alinity i Estradiol Reagent Kit, catalogue number 07P50 (Abbott Laboratories, Abbott Park, IL, USA). The assay is based on chemiluminescent microparticle immunoassay technology for quantitative determination of estradiol in serum and plasma, and results were reported in pmol/L. The analytical measuring interval was 88–3670 pmol/L, the lower limit of detection was 73 pmol/L, and the limit of quantification was 88 pmol/L. The within-run coefficients of variation were 7.2%, 2.4%, and 2.2%, and the within-laboratory total coefficients of variation were 7.7%, 2.6%, and 2.6% at estradiol concentrations of 180, 688, and 2156 pmol/L, respectively. Laboratory reference intervals were phase- and menopausal-status specific; because these reproductive-stage variables were unavailable in the analytical dataset, reference intervals were reported for laboratory description only and were not used to define the study outcome.

Information on timing of blood sampling in relation to the menstrual cycle, fasting duration at venipuncture, pregnancy and lactation status, menopausal status, hormonal contraception, menopausal hormone therapy, and previous ovarian surgery was not available in the working analytical extract.

Accordingly, serum estradiol values were considered as single cross-sectional biochemical measurements within a population survey framework. Their interpretation was therefore limited to distributional and associative analyses rather than individual-level assessment of ovarian function or menopausal status.

### 2.4. Outcome and Covariates

The primary binary outcome for association analyses was a lower-tail estradiol category defined as E2 < 200 pmol/L. This cutoff corresponded approximately to the lower quartile of the analytical female subsample and was used to identify the lower part of the observed estradiol distribution. Because the working dataset did not contain menstrual-cycle phase, menopausal status, hormonal medication use, pregnancy or lactation status, this threshold was treated as an analytical distributional category rather than a clinical reproductive diagnosis.

Age was categorized as 18–29, 30–39, 40–49, 50–59, and 60–69 years, consistent with commonly used age stratifications in epidemiological studies [17]. BMI was categorized as underweight (<18.5 kg/m^2^), normal weight (18.5–24.9 kg/m^2^), overweight (25.0–29.9 kg/m^2^), and obesity (≥30.0 kg/m^2^) [18].

Residence was coded as urban or rural. Ethnicity was grouped as Kazakh, Russian, and other. Marital status was collapsed into married/in civil union, single, and separated/divorced/widowed. Education was categorized as higher/postgraduate, technical or vocational, completed secondary, and basic secondary or less. Smoking status was analyzed as current smoking yes/no. Alcohol consumption was grouped as rare (<1/month or holidays), 1–3 days/month, and ≥1–2 days/week.

HbA1c was categorized as normal (<5.7%), prediabetes (5.7–6.4%), and diabetes (≥6.5%) according to American Diabetes Association diagnostic criteria [19]. Triglycerides were dichotomized as <1.7 versus ≥1.7 mmol/L, LDL cholesterol as <3.0 versus ≥3.0 mmol/L, and HDL cholesterol as low versus normal/high. Low HDL cholesterol was defined as HDL cholesterol < 1.3 mmol/L in women, consistent with commonly used metabolic-risk criteria [20].

### 2.5. Statistical Analysis

Descriptive statistics are presented as mean +/− standard deviation, median with interquartile range, or number with percentage, as appropriate. Tables report unweighted analytical counts and percentages to maintain transparency between denominators and reported percentages. The sampling design is described to define the source population and survey framework; regression analyses were performed as complete-case analytical models for women with available estradiol and covariate data. Because the present analysis used an analytical subsample with complete estradiol and covariate data, estimates should be interpreted as analytical-sample estimates rather than fully weighted national prevalence estimates.

Assay-specific analytical characteristics, including the analytical measuring interval, lower limit of detection, limit of quantification, and coefficients of variation, were documented in the Section 2.3. The primary E2 < 200 pmol/L threshold was above the assay limit of quantification and was used as an analytical lower-tail category rather than as a clinical diagnostic threshold.

Associations with the lower-tail estradiol category were examined using logistic regression models and are reported as adjusted odds ratios (aORs) with 95% confidence intervals (95% CIs) in the main table. Serum estradiol was also analyzed as a continuous outcome on the logarithmic scale using ordinary least squares regression, with back-transformed coefficients expressed as percentage differences.

Age-specific distributional summaries were used to display estradiol values across age groups. The age-specific table reports P25, P50, and P75 because these quartiles were available consistently from the analytical summaries and are directly aligned with the main lower-tail threshold. These quartiles were treated as analytical distributional summaries and not as clinical reference intervals.

Potential nonlinear relationships were explored in a diagnostic logistic regression model for the lower-tail estradiol outcome, defined as E2 < 200 pmol/L. Continuous age, BMI, and HbA1c were included as restricted cubic spline terms with four degrees of freedom. The spline model was compared with a corresponding model in which these predictors were entered as linear continuous terms, using the likelihood-ratio test and model-fit indices, including the Akaike information criterion (AIC), McFadden pseudo-R2, Brier score, and area under the ROC curve (AUC). This comparison assessed the joint contribution of nonlinear terms for the continuous predictors. Regional variation in estradiol was assessed descriptively using unweighted and unadjusted analytical-sample comparisons, including one-way analysis of variance, the Kruskal–Wallis test, and the intraclass correlation coefficient (ICC) for ln(E2). Descriptive age-window observations were used to show the distribution of lower-tail estradiol among women aged 18–39 years and non-lower-tail estradiol among women aged 50–69 years. Sensitivity analyses examined alternative thresholds of <150 and <250 pmol/L. Two-sided *p*-values < 0.05 were considered statistically significant.

## 3. Results

### 3.1. Study Population and Estradiol Distribution

Of the parent survey sample, 3498 women with serum estradiol measurements were available for the present analysis. The mean age of the analytical sample was 43.21 +/− 13.74 years, and the median serum estradiol concentration was 313.00 pmol/L (interquartile range 207.25–457.00). Most women lived in urban areas (65.4%), 60.6% had higher or postgraduate education, and 60.8% were married or living in a civil union (Table 1).

The age distribution was broad, with 20.0% of women aged 18–29 years, 22.0% aged 30–39 years, 22.2% aged 40–49 years, 20.4% aged 50–59 years, and 15.4% aged 60–69 years. Overweight and obesity were common, affecting 32.1% and 23.3% of the sample, respectively. Current smoking was reported by 11.5% of participants, while 27.9% had low HDL cholesterol.

### 3.2. Age-Specific Estradiol Distribution

**Figure 2:**
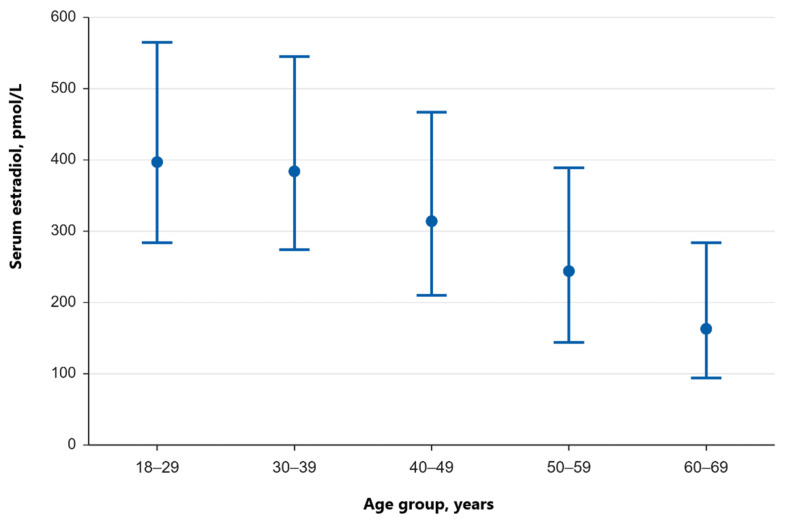
Age-specific median and interquartile range of serum estradiol in the analytical sample of women from Kazakhstan.

Estradiol distribution differed substantially by age group (Table 2; Figure 2). Median E2 values were highest in younger adult women and declined progressively in older age groups. The proportion of women below 200 pmol/L increased most clearly after the age of 40 years and was highest among women aged 60–69 years. This pattern is consistent with the expected influence of reproductive aging, although exact menopausal status was not available in the working dataset.

Because menopausal status was not recorded, these age-specific patterns should not be interpreted as direct comparisons between premenopausal and postmenopausal women. Rather, they describe the distribution of estradiol across age-defined epidemiological strata. This distinction is important because estradiol varies substantially during the menstrual cycle in reproductive-age women and remains generally low after menopause, whereas a single cross-sectional measurement cannot distinguish these physiological states.

The age-specific distributional display also shows that the lower-tail category was not confined to older women. Among women aged 18–39 years, 165 of 1469 women (11.2%) had E2 < 200 pmol/L. Conversely, among women aged 50–69 years, 730 of 1252 women (58.3%) had E2 ≥ 200 pmol/L. These observations support a cautious distributional interpretation rather than a diagnostic interpretation of the threshold.

### 3.3. Regional Variation and Geographic Pattern

In the analytical sample, mean serum estradiol was 378.42 pmol/L (95% CI 366.24–390.60). In unweighted and unadjusted analytical-sample comparisons, regional mean values varied numerically, but there was little evidence of statistically meaningful between-region heterogeneity (Table 3). Neither one-way ANOVA (F [19, 3478] = 0.90, *p* = 0.583) nor the Kruskal–Wallis test (H = 21.95, *p* = 0.287) indicated significant regional differences, and the effect size was minimal (eta-squared = 0.0049). Consistently, the ICC for ln(E2) was 0.000861, indicating that only a negligible proportion of variance was attributable to the regional level.

These findings argue against a stable geographic clustering pattern in this analytical sample. In practical terms, the regional signal was much weaker than the individual-level age and metabolic signal. The main analytical focus was therefore placed on age, adiposity, and HDL cholesterol rather than on region-specific hormonal zones.

### 3.4. Factors Associated with the Lower-Tail Estradiol Category

**Figure 3:**
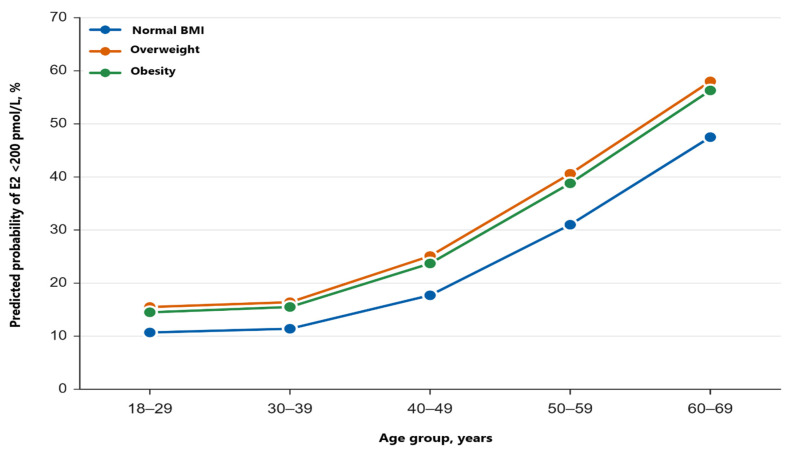
Adjusted predicted probability of the lower-tail estradiol category (E2 < 200 pmol/L) by age group and BMI category in the analytical sample.

In the main binary-outcome analysis, 837 women (23.9%) belonged to the lower-tail estradiol category defined as E2 < 200 pmol/L. In the multivariable logistic model, age demonstrated a marked non-linear pattern (Table 4; Figure 3). Compared with women aged 18–29 years, the adjusted odds of belonging to the lower-tail estradiol category were not significantly different in women aged 30–39 years, increased in women aged 40–49 years, and rose further in women aged 50–59 and 60–69 years.

Among anthropometric variables, overweight and obesity were associated with higher odds of belonging to the lower-tail estradiol category after multivariable adjustment. Low HDL cholesterol remained independently associated with the lower-tail estradiol category. Residence, education, ethnicity, smoking, alcohol use, HbA1c category, triglycerides, and LDL cholesterol were not statistically significant in the fully adjusted model. Overall discrimination of the main multivariable model was moderate (AUC 0.742; Brier score 0.148; McFadden pseudo-R2 0.151; AIC 3289.4).

### 3.5. Continuous-Outcome Analysis and Additional Analyses

When estradiol was analyzed as a continuous outcome on the logarithmic scale, several of the main patterns remained visible, although the effect metric differed (Table 5). Relative to women aged 18–29 years, estradiol levels on the multiplicative scale were lower in women aged 40–49, 50–59, and 60–69 years. Overweight and obesity were also associated with lower estradiol levels. Low HDL cholesterol was associated with a 5.9% lower estradiol level (95% CI −10.5% to −1.0%).

In the diagnostic logistic model for the lower-tail estradiol outcome, defined as E2 < 200 pmol/L, continuous age, BMI, and HbA1c were modeled using restricted cubic splines with four degrees of freedom. Compared with the corresponding model in which these predictors were entered as linear continuous terms, the spline model showed better fit (AIC 3666.4 vs. 3846.2; McFadden pseudo-R2 0.053 vs. 0.003; AUC 0.657 vs. 0.545; likelihood-ratio *p* < 0.001). This comparison evaluated the joint contribution of nonlinear terms for the continuous predictors and was used as a diagnostic analysis rather than as a replacement for the categorical multivariable model shown in Table 4. Formal interaction terms for age × BMI (*p* = 0.204) and BMI × HbA1c (*p* = 0.238) were not statistically significant. Sensitivity analyses using alternative lower-tail thresholds of E2 < 150 pmol/L and E2 < 250 pmol/L are presented in Supplementary Table S1. Across alternative thresholds, older age groups remained strongly associated with lower-tail estradiol, and overweight and obesity showed broadly consistent associations. The association with low HDL cholesterol was weaker at the alternative thresholds, supporting cautious interpretation of threshold-based categorizations.

## 4. Discussion

This study describes serum estradiol distribution in a national survey-based analytical sample of adult women in Kazakhstan and shows that the lower tail of the E2 distribution was associated primarily with age-metabolic characteristics rather than regional differences. The main finding is that the lower-tail E2 profile was predominantly associated with age, BMI, and HDL cholesterol. At the same time, there was little evidence of regional clustering in this analytical sample, indicating that the observed profile was more strongly related to individual-level characteristics than to region.

Given the cross-sectional design and the use of a single estradiol measurement, all findings should be interpreted as associations rather than causal, diagnostic, or screening-related evidence.

Age was the strongest factor associated with the lower-tail estradiol category. Compared with women aged 18–29 years, the adjusted odds of E2 < 200 pmol/L increased progressively in the 40–49, 50–59, and 60–69 year groups. A similar trend was also observed when E2 was analyzed as a continuous outcome. These findings are broadly consistent with reproductive aging and the menopause transition, during which declining estrogen activity is a central biological feature and hormonal changes may extend over several years [5]. This interpretation, however, should remain cautious. The strong age-related association may partly reflect menopausal transition and postmenopausal status, but these reproductive stages were not directly documented in the survey. Therefore, age should be regarded as an approximate epidemiological marker of reproductive-stage heterogeneity rather than a confirmed clinical classification of menopausal status.

However, the age-related findings require careful reproductive-physiological interpretation. In women, serum estradiol is highly dynamic during the menstrual cycle and differs substantially between reproductive-age, perimenopausal, and postmenopausal states [10,21,22]. In our analytical dataset, information on menstrual-cycle timing, menopausal status, pregnancy and lactation, hormonal contraception, menopausal hormone therapy, and previous ovarian surgery was unavailable. Consequently, the age groups used in this study should not be treated as clinical reproductive-stage categories. This is particularly important because some women aged 18–39 years had E2 < 200 pmol/L, whereas more than half of women aged 50–69 years had E2 ≥ 200 pmol/L. Therefore, the selected threshold reflects the lower part of the observed estradiol distribution rather than a diagnosis of hypoestrogenism, menopause, or impaired ovarian function. Thus, the study does not distinguish physiological cyclic estradiol variability, the menopausal transition, postmenopausal estrogen levels, and clinically low estrogen states. Taken together, these results describe an age- and metabolism-associated distribution of lower-tail estradiol within a national survey-based analytical sample, without implying clinical classification at the individual level.

Another observed association was the relationship between E2 < 200 pmol/L and overweight or obesity. In the adjusted model, overweight and obesity were associated with higher odds of belonging to the lower-tail estradiol category, while in the continuous-outcome analysis they were associated with lower E2 levels. These findings are consistent with current concepts that adipose tissue is an active endocrine organ involved in the regulation of estrogen metabolism, inflammation, and metabolic function [23,24,25].

At the same time, the association between BMI and estradiol should not be interpreted simplistically. Adipose tissue may participate in the peripheral metabolism of estrogens, but the direction of the association may differ depending on age and reproductive status [26]. In our study, the absence of menopausal-status data did not allow separation of premenopausal, perimenopausal, and postmenopausal women. Therefore, the BMI-related findings should primarily be interpreted as part of a broader age-metabolic profile rather than evidence of a single biological mechanism across all age groups. This interpretation is consistent with evidence that the menopausal transition and adiposity are associated with changes in cardiometabolic risk [27] and lipid metabolism [3].

We also assessed the age × BMI interaction, which was not statistically significant. However, this finding should be interpreted cautiously because age strata are only epidemiological approximations and do not replace clinically defined menopausal or reproductive status. Therefore, the BMI-E2 association should not be interpreted as evidence of a uniform biological mechanism across all women.

Low HDL cholesterol also showed a modest association with the lower-tail estradiol category and with lower estradiol values in the continuous-outcome analysis. This finding should be interpreted within the broader age-metabolic context of the study. Estradiol measures and HDL cholesterol may co-vary across the female life course through overlapping influences of age, adiposity, reproductive-stage heterogeneity, lifestyle, treatment, and other metabolic factors. Given the cross-sectional design and the absence of menopausal-status data, the observed association should not be interpreted as evidence of a directional effect between estradiol and HDL cholesterol. Rather, it suggests that low HDL may be one component of the metabolic profile that co-occurs with lower-tail estradiol in this analytical sample. This interpretation is consistent with literature describing links between estradiol measures, HDL function, and age-related lipid metabolism in women [28].

Among the metabolic indicators, the most consistent associations were observed for BMI and HDL cholesterol. HbA1c, LDL cholesterol, and triglycerides did not retain stable independent associations in the fully adjusted model. This may reflect the fact that these indicators are influenced by a broad clinical and behavioral context, including adiposity, diet, physical activity, treatment, and comorbid conditions [29,30]. Therefore, their absence as independent predictors should not be interpreted as evidence of the absence of a biological relationship. Rather, within this model, they did not provide additional explanatory information after adjustment for age, BMI, and HDL cholesterol, which may be related to overlap among metabolic factors and correlation between covariates [31].

In this analytical sample, the limited evidence of regional clustering was an important descriptive finding. Despite numerical differences in mean E2 values across regions of Kazakhstan, statistically significant interregional differences were not confirmed, and the ICC for ln(E2) was extremely low. This indicates that the regional level explained only a minimal proportion of estradiol variability [32]. Nevertheless, crude regional summaries may still be influenced by regional differences in age and metabolic composition. For a country with substantial geographic and sociodemographic diversity, this finding is important because it does not support the concept of stable regional hormonal patterns. It is more likely that differences in E2 distribution were related to the age and metabolic composition of women rather than to the region of residence itself.

## 5. Strengths and Limitations

The main strengths of this study include the large national survey-based analytical sample of women, standardized data collection, biochemical measurement of serum estradiol, age-specific distributional presentation, and complementary analytical approaches, including descriptive, regression-based, nonlinearity, regional-heterogeneity, and sensitivity analyses. The study also provides a useful negative finding: regional clustering was negligible, which helps prevent overinterpretation of numeric regional differences.

The main limitations are the cross-sectional design, which precludes causal inference, and the use of a single estradiol measurement, which cannot capture within-person cyclic variation. The working dataset did not retain information on menstrual-cycle timing, menopausal status, pregnancy and lactation, hormonal contraception, menopausal hormone therapy, or prior ovarian surgery. As a result, we were unable to stratify women into premenopausal, perimenopausal, and postmenopausal groups, which would have provided a more clinically precise framework for estradiol interpretation. Age-group analyses were therefore used as an epidemiological approximation and should not be considered a substitute for clinically defined reproductive status. These omissions constrain biological interpretation and may have contributed to residual heterogeneity in the age-specific pattern. In addition, although assay-specific analytical characteristics were documented, the study relied on a single estradiol measurement and did not include repeated hormone testing. Finally, the threshold used in this study identifies the lower part of the observed sample distribution and should not be used to define clinical hypoestrogenism.

## 6. Conclusions

In this large national survey-based analytical sample of adult women in Kazakhstan, serum estradiol showed broad inter-individual variation and negligible regional clustering. The lower-tail estradiol category was more closely characterized by age, adiposity, and HDL cholesterol than by geography. Future studies should incorporate menstrual-cycle timing, menopausal status, hormonal medication use, pregnancy and lactation status, and detailed assay documentation to improve biological and clinical interpretation. These findings describe analytical-sample associations and do not support individual clinical classification or screening based on a single estradiol measurement.

## Figures and Tables

**Table 1:** Characteristics of women in the analytical sample with measured serum estradiol (n = 3498).

Characteristic	Women
Age, years	43.21 +/− 13.74
Age group, *n* (%)	
18–29	698 (20.0%)
30–39	771 (22.0%)
40–49	777 (22.2%)
50–59	714 (20.4%)
60–69	538 (15.4%)
Body mass index, kg/m^2^	26.43 +/− 5.57
BMI category, *n* (%)	
Underweight (<18.5)	160 (4.6%)
Normal weight (18.5–24.9)	1401 (40.1%)
Overweight (25.0–29.9)	1123 (32.1%)
Obesity (≥30.0)	814 (23.3%)
Serum estradiol, pmol/L	313.00 [207.25; 457.00]
Lower-tail estradiol category, E2 < 200 pmol/L	837 (23.9%)
Place of residence, *n* (%)	
Urban	2289 (65.4%)
Rural	1209 (34.6%)
Education, *n* (%)	
Higher education or postgraduate	2120 (60.6%)
Technical/vocational	1079 (30.8%)
Completed secondary	225 (6.4%)
Basic secondary or less	74 (2.1%)
Ethnicity, *n* (%)	
Kazakh	2489 (71.2%)
Russian	624 (17.8%)
Other	385 (11.0%)
Marital status, *n* (%)	
Married/in civil union	2127 (60.8%)
Single	717 (20.5%)
Separated/divorced/widowed	654 (18.7%)
Current smoking, *n* (%)	
No	3096 (88.5%)
Yes	402 (11.5%)
Alcohol consumption during previous 12 months, *n* (%)	
Rare (<1/month or holidays)	3069 (87.7%)
1–3 days/month	167 (4.8%)
≥1–2 days/week	262 (7.5%)
Glycemic status by HbA1c, *n* (%)	
Normal (<5.7%)	2814 (80.4%)
Prediabetes (5.7–6.4%)	532 (15.2%)
Diabetes (≥6.5%)	152 (4.3%)
Triglycerides, *n* (%)	
<1.7 mmol/L	2905 (83.0%)
≥1.7 mmol/L	593 (17.0%)
LDL cholesterol, *n* (%)	
<3.0 mmol/L	1954 (55.9%)
≥3.0 mmol/L	1544 (44.1%)
HDL cholesterol, *n* (%)	
Normal/high	2522 (72.1%)
Low	976 (27.9%)

**Table 2:** Age-specific analytical quartiles of serum estradiol.

Age Group, Years	*n*	P25, pmol/L	P50, pmol/L	P75, pmol/L	E2 < 200 pmol/L, *n*/N (%)
18–29	698	285.00	398.00	565.00	75/698 (10.7%)
30–39	771	275.00	385.00	545.00	90/771 (11.7%)
40–49	777	210.00	315.00	470.00	150/777 (19.3%)
50–59	714	145.00	245.00	390.00	240/714 (33.6%)
60–69	538	95.00	165.00	285.00	282/538 (52.4%)
Total	3498	207.25	313.00	457.00	837/3498 (23.9%)

**Table 3:** Unweighted regional distribution of serum estradiol in the analytical sample.

Region	*n*	Mean E2, pmol/L	95% CI	Median E2 [IQR], pmol/L
Astana city	271	428.21	370.76–485.67	337.00 [179.50; 641.00]
Almaty city	422	386.75	349.11–424.39	285.00 [152.00; 530.50]
Shymkent city	223	350.58	321.58–379.58	301.50 [173.50; 511.25]
Akmola Region	136	353.69	311.54–395.84	346.00 [190.25; 454.75]
Aktobe Region	156	352.38	316.43–388.33	259.00 [135.00; 525.50]
Almaty Region	263	358.19	316.88–399.50	322.00 [159.50; 489.50]
Atyrau Region	136	403.60	326.12–481.07	338.00 [180.50; 516.75]
West Kazakhstan Region	119	380.89	289.92–471.87	270.00 [131.00; 446.00]
Zhambyl Region	214	375.75	342.09–409.41	389.00 [180.50; 604.25]
Karaganda Region	203	364.11	306.24–421.99	282.00 [161.00; 454.00]
Kostanay Region	155	354.02	311.68–396.36	270.00 [135.00; 506.50]
Kyzylorda Region	144	400.96	304.96–496.96	305.00 [143.50; 506.50]
Mangystau Region	126	406.99	318.82–495.16	286.50 [146.00; 533.25]
Turkistan Region	303	364.26	324.21–404.31	345.00 [182.75; 513.50]
Pavlodar Region	141	424.96	360.14–489.79	330.00 [137.00; 666.00]
North Kazakhstan Region	97	409.80	352.43–467.17	369.00 [185.50; 608.50]
East Kazakhstan Region	130	332.62	301.72–363.52	285.50 [189.75; 437.00]
Abai Region	104	372.02	324.96–419.07	333.00 [173.00; 419.00]
Zhetysu Region	112	379.96	328.59–431.32	376.00 [157.25; 555.75]
Ulytau Region	43	373.03	304.72–441.35	365.50 [184.75; 509.00]

**Table 4:** Adjusted associations with a lower-tail estradiol category (E2 < 200 pmol/L).

Variable	Category	E2 < 200, *n*/N (%)	aOR (95% CI)	*p*
Age, years	18–29	75/698 (10.7%)	1.00 (ref)	-
	30–39	90/771 (11.7%)	1.08 (0.78–1.48)	0.650
	40–49	150/777 (19.3%)	1.82 (1.31–2.53)	<0.001
	50–59	240/714 (33.6%)	3.72 (2.60–5.33)	<0.001
	60–69	282/538 (52.4%)	7.48 (5.23–10.70)	<0.001
BMI category	Underweight	45/160 (28.1%)	1.41 (0.93–2.15)	0.106
	Normal	250/1401 (17.8%)	1.00 (ref)	-
	Overweight	320/1123 (28.5%)	1.53 (1.23–1.90)	<0.001
	Obesity	222/814 (27.3%)	1.42 (1.11–1.82)	0.006
Place of residence	Urban	552/2289 (24.1%)	1.00 (ref)	-
	Rural	285/1209 (23.6%)	0.97 (0.81–1.15)	0.698
Education	Higher/postgraduate	521/2120 (24.6%)	1.00 (ref)	-
	Technical/vocational	243/1079 (22.5%)	0.86 (0.72–1.04)	0.117
	Completed secondary	58/225 (25.8%)	0.95 (0.67–1.34)	0.778
	Basic secondary or less	15/74 (20.3%)	0.71 (0.39–1.28)	0.250
Ethnicity	Kazakh	601/2489 (24.1%)	1.00 (ref)	-
	Russian	137/624 (22.0%)	0.92 (0.73–1.16)	0.470
	Other	99/385 (25.7%)	1.20 (0.92–1.57)	0.172
Marital status	Married/civil union	474/2127 (22.3%)	1.00 (ref)	-
	Single	204/717 (28.5%)	1.25 (0.99–1.57)	0.062
	Separated/divorced/widowed	159/654 (24.3%)	1.16 (0.93–1.45)	0.185
Current smoking	No	754/3096 (24.4%)	1.00 (ref)	-
	Yes	83/402 (20.6%)	0.88 (0.66–1.16)	0.361
Alcohol use	Rare	744/3069 (24.2%)	1.00 (ref)	-
	1–3 days/month	40/167 (24.0%)	1.18 (0.78–1.77)	0.437
	≥1–2 days/week	53/262 (20.2%)	0.97 (0.68–1.37)	0.844
HbA1c category	Normal (<5.7%)	685/2814 (24.3%)	1.00 (ref)	-
	Prediabetes (5.7–6.4%)	112/532 (21.1%)	0.89 (0.70–1.14)	0.352
	Diabetes (≥6.5%)	40/152 (26.3%)	1.11 (0.76–1.63)	0.583
Triglycerides	<1.7 mmol/L	715/2905 (24.6%)	1.00 (ref)	-
	≥1.7 mmol/L	122/593 (20.6%)	0.78 (0.61–1.00)	0.052
LDL cholesterol	<3.0 mmol/L	510/1954 (26.1%)	1.00 (ref)	-
	≥3.0 mmol/L	327/1544 (21.2%)	0.86 (0.71–1.03)	0.095
HDL cholesterol	Normal/high	578/2522 (22.9%)	1.00 (ref)	-
	Low	259/976 (26.5%)	1.26 (1.04–1.53)	0.019

**Table 5:** Predictors of serum estradiol as a continuous outcome (OLS for ln[E2]).

Term	Delta E2, % (95% CI)	*p*
Age 30–39 years	−3.8% (−11.7% to 4.8%)	0.378
Age 40–49 years	−12.6% (−20.4% to −4.1%)	0.004
Age 50–59 years	−32.4% (−38.6% to −25.6%)	<0.001
Age 60–69 years	−45.8% (−51.2% to −39.8%)	<0.001
BMI: obesity (≥30.0)	−10.7% (−15.6% to −5.5%)	<0.001
BMI: overweight (25.0–29.9)	−7.9% (−12.2% to −3.4%)	0.001
BMI: underweight (<18.5)	−2.5% (−13.5% to 9.9%)	0.675
Rural residence	1.0% (−3.5% to 5.7%)	0.676
Education: completed secondary	6.2% (−3.6% to 16.9%)	0.221
Education: technical/vocational	4.5% (−0.3% to 9.5%)	0.065
Education: basic secondary or less	7.9% (−6.5% to 24.4%)	0.299
Ethnicity: other	−5.6% (−11.8% to 1.0%)	0.094
Ethnicity: Russian	2.0% (−3.6% to 7.9%)	0.501
Marital status: separated/divorced/widowed	−6.1% (−11.1% to −0.9%)	0.023
Marital status: single	−2.6% (−8.9% to 4.1%)	0.433
Currently smoking	2.9% (−3.9% to 10.1%)	0.412
Alcohol: 1–3 days/month	−2.4% (−12.0% to 8.3%)	0.653
Alcohol: ≥1–2 days/week	1.4% (−6.7% to 10.3%)	0.742
HbA1c: diabetes (≥6.5%)	−9.0% (−17.5% to 0.3%)	0.057
HbA1c: prediabetes (5.7–6.4%)	−2.9% (−8.0% to 2.6%)	0.295
Triglycerides ≥ 1.7 mmol/L	1.0% (−4.7% to 7.0%)	0.736
LDL cholesterol ≥ 3.0 mmol/L	0.9% (−3.8% to 5.7%)	0.724
Low HDL cholesterol	−5.9% (−10.5% to −1.0%)	0.018

## Data Availability

All available data were presented within the manuscript.

## Supplementary Materials

The following supporting information can be downloaded at: https://www.mdpi.com/article/10.3390/ijerph23070934/s1, Table S1: Sensitivity analysis using alternative lower-tail estradiol thresholds.

## Abbreviations

The following abbreviations are used in this manuscript:

E2	Estradiol
WHO	World Health Organization
STEPS	STEPwise Approach to Noncommunicable Disease Risk Factor Surveillance
BMI	Body Mass Index
HDL	High-Density Lipoprotein
LDL	Low-Density Lipoprotein
HbA1c	Glycated Hemoglobin
ANOVA	Analysis of Variance
ICC	Intraclass Correlation Coefficient
aOR	Adjusted Odds Ratio
CI	Confidence Interval
AUC	Area Under the Curve
AIC	Akaike Information Criterion
OLS	Ordinary Least Squares
ROC	Receiver Operating Characteristic
IQR	Interquartile Range
SD	Standard Deviation
CMIA	Chemiluminescent Microparticle Immunoassay
NCD	Noncommunicable Disease
