# Supplementary material for: Age-Metabolic Profile of Lower Serum Estradiol Among Women in Kazakhstan: A Large National Survey-Based Analytical Study
Source: Int J Environ Res Public Health. 2026 Jul 21;23(7):934. doi: 10.3390/ijerph23070934 (PMC13411955; doi:10.3390/ijerph23070934)
Supplement: Supplementary file 1 [file ijerph-23-00934-s001.zip › ijerph-4400151-supplementary.pdf]

Supplementary Table S1. Sensitivity analysis using alternative lower-tail estradiol thresholds.

**Threshold**	**Women below threshold, n/N (%)**	**Age 40-49 aOR (95% CI)**	**Age 50-59 aOR (95% CI)**	**Age 60-69 aOR (95% CI)**	**Overweight aOR (95% CI)**	**Obesity aOR (95% CI)**	**Low HDL aOR (95% CI)**
E2 <150 pmol/L	520/3498 (14.9%)	2.10 (1.38-3.19)	5.55 (3.71-8.31)	13.80 (9.20-20.70)	1.42 (1.08-1.87)	1.35 (1.00-1.82)	1.21 (0.96-1.53)
E2 <200 pmol/L	837/3498 (23.9%)	1.82 (1.31-2.53)	3.72 (2.60-5.33)	7.48 (5.23-10.70)	1.53 (1.23-1.90)	1.42 (1.11-1.82)	1.26 (1.04-1.53)
E2 <250 pmol/L	1254/3498 (35.8%)	1.62 (1.23-2.13)	2.90 (2.17-3.88)	5.62 (4.13-7.65)	1.43 (1.18-1.74)	1.34 (1.08-1.66)	1.18 (0.99-1.41)

Reference categories: age 18-29 years, normal BMI, and normal/high HDL cholesterol.
